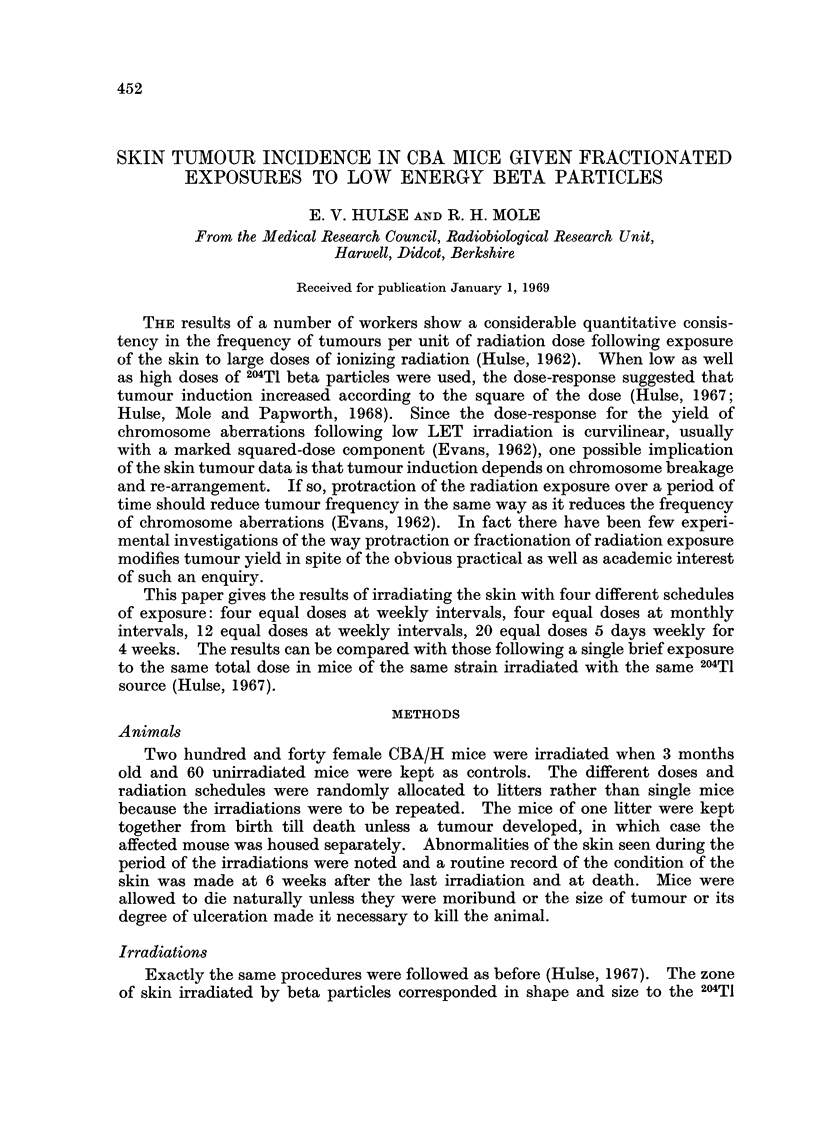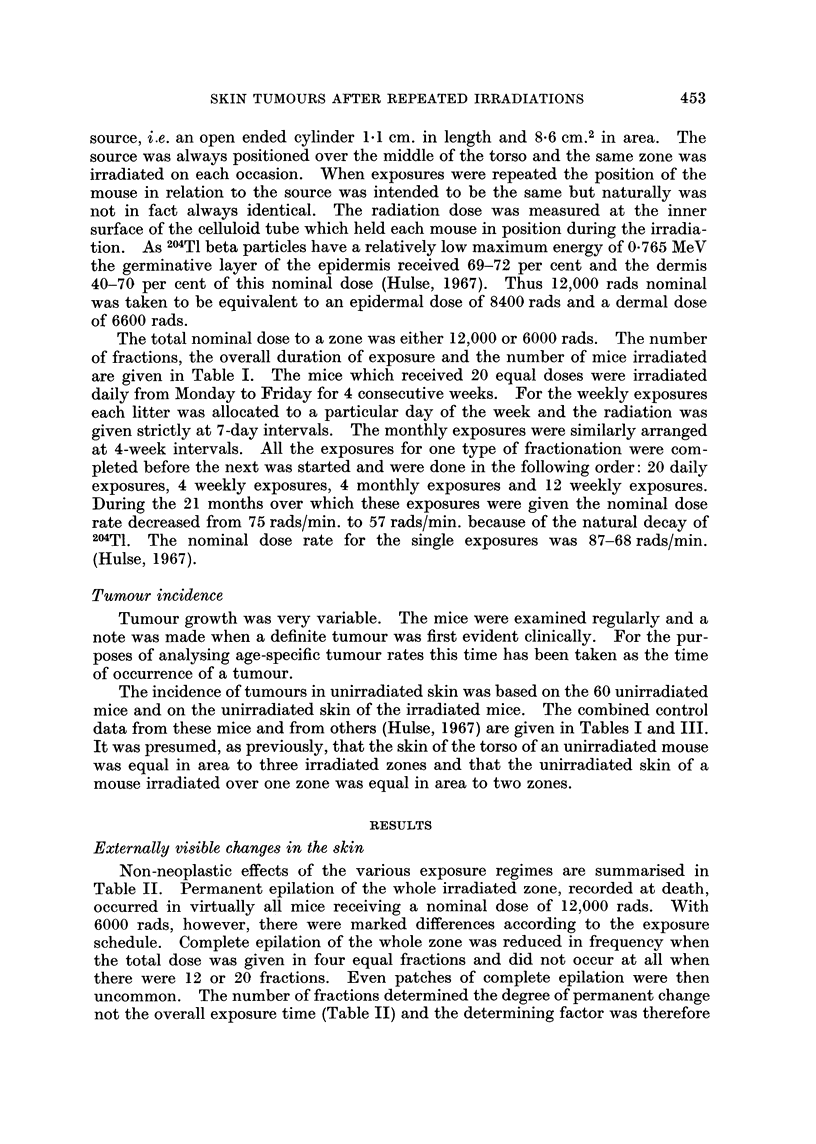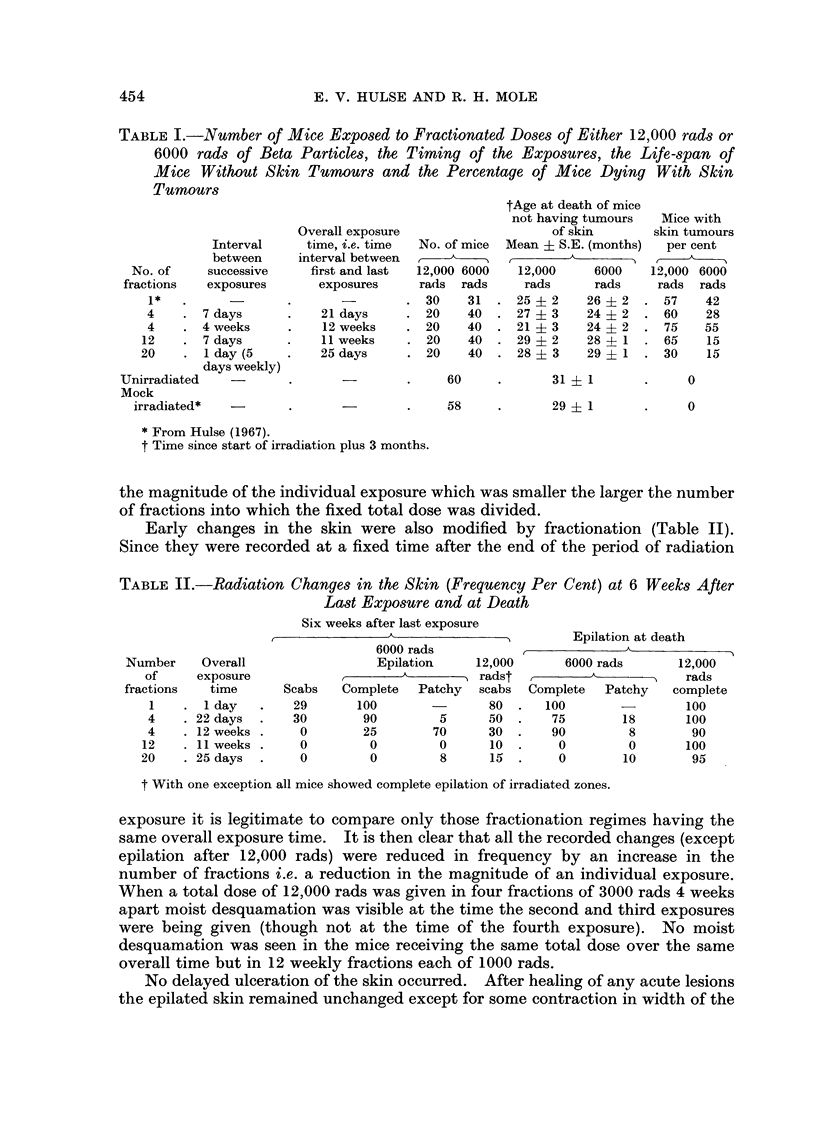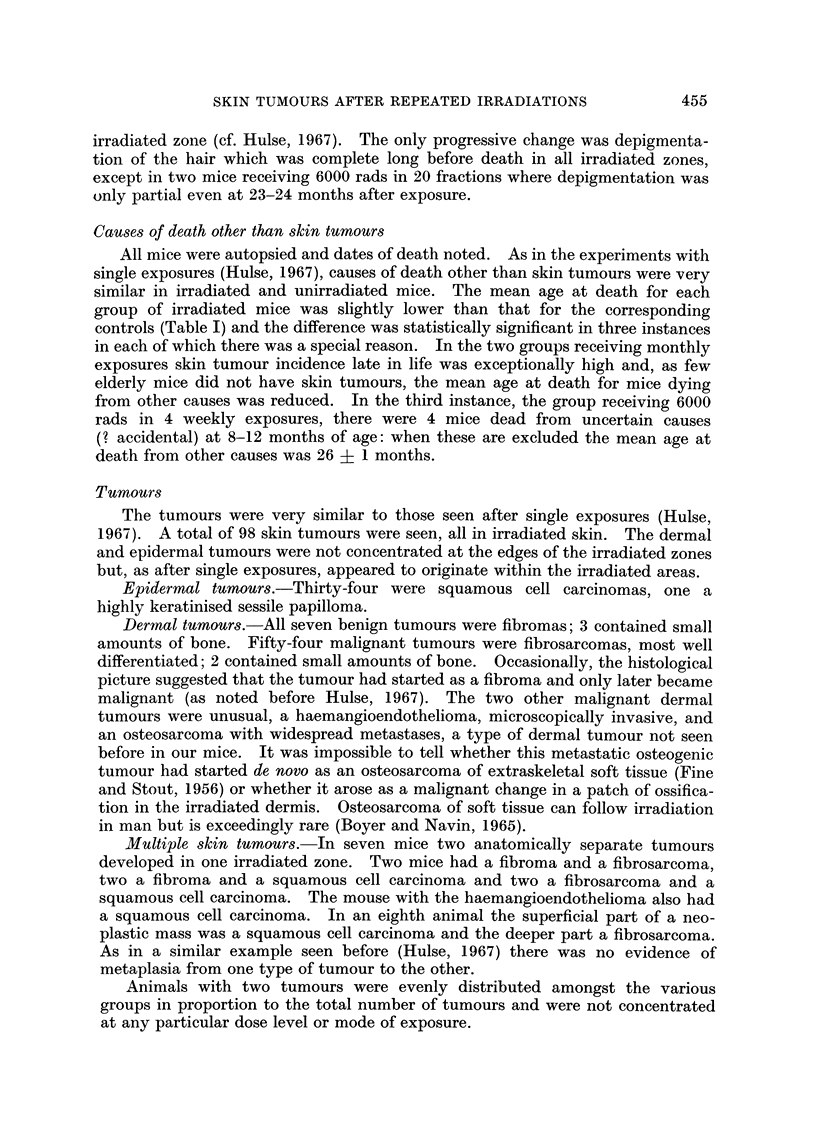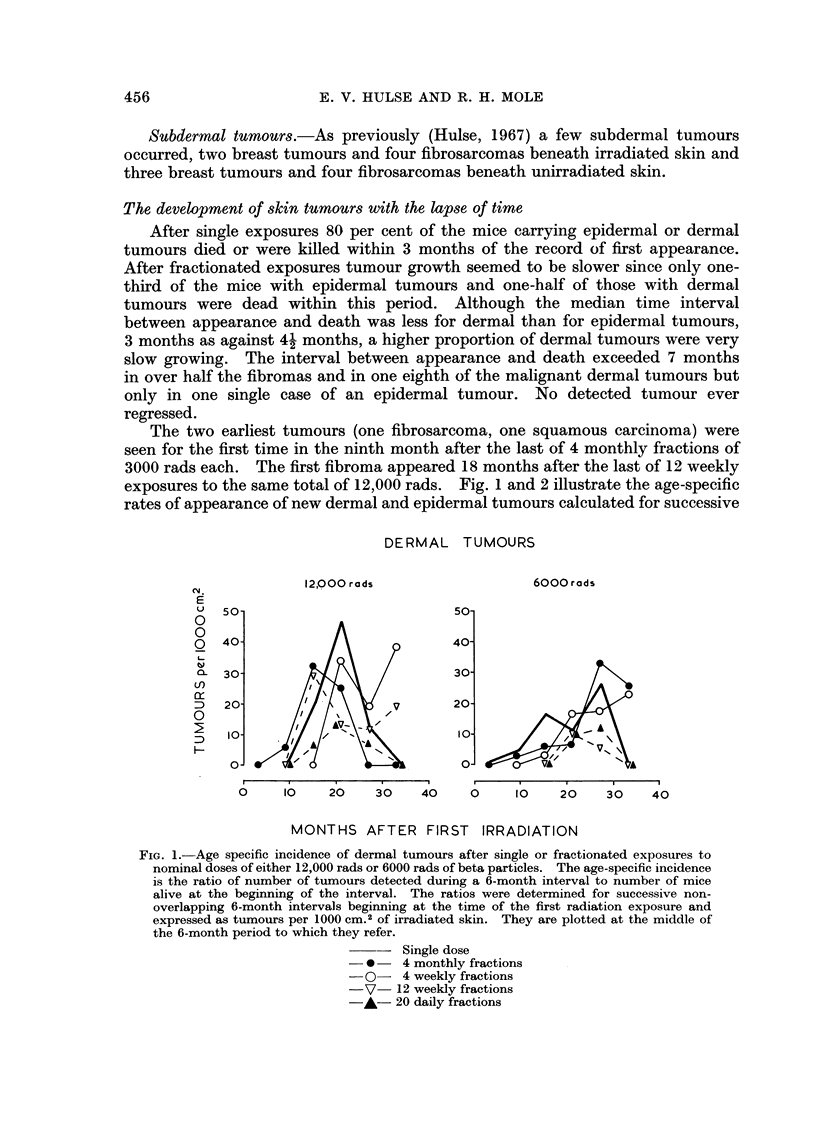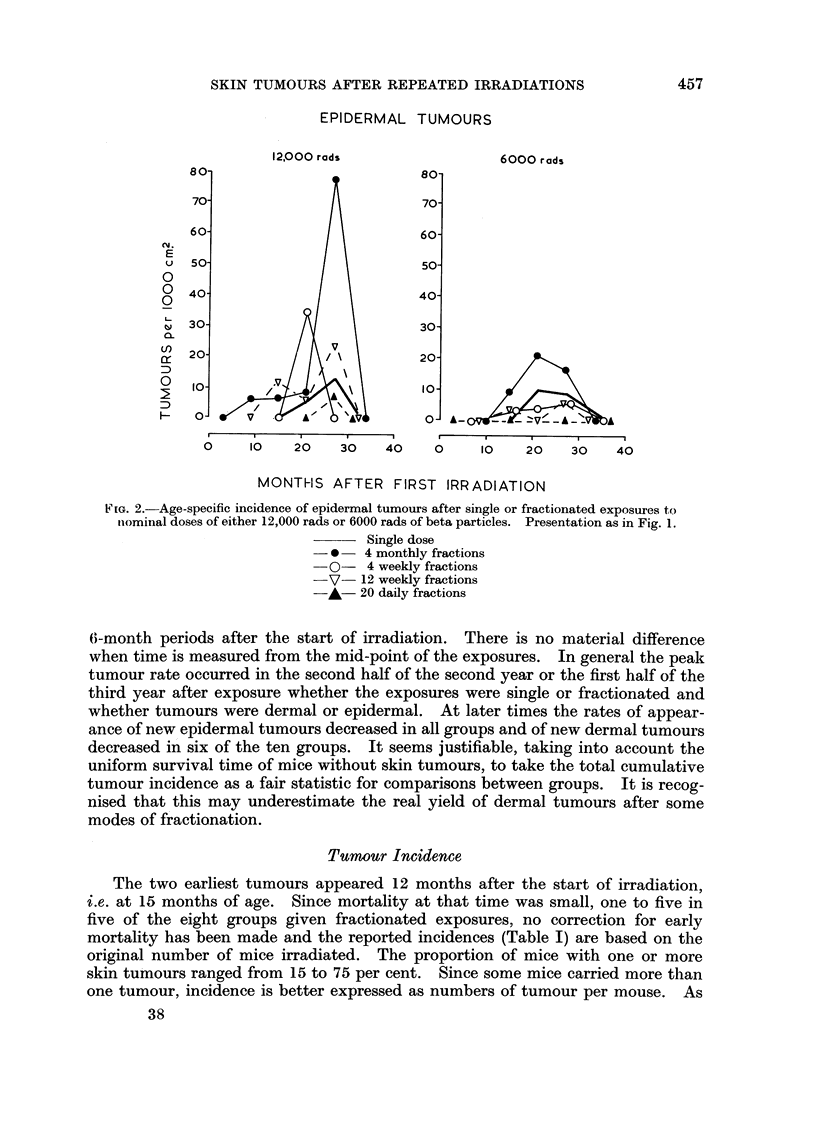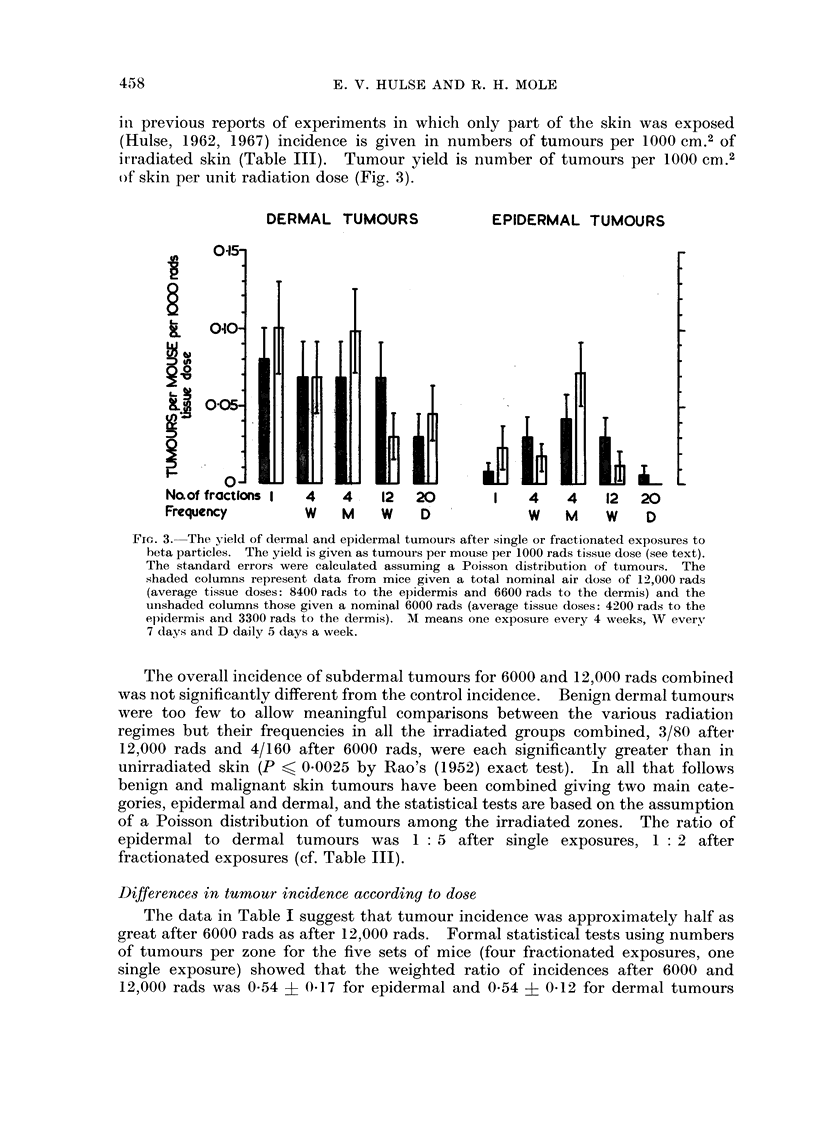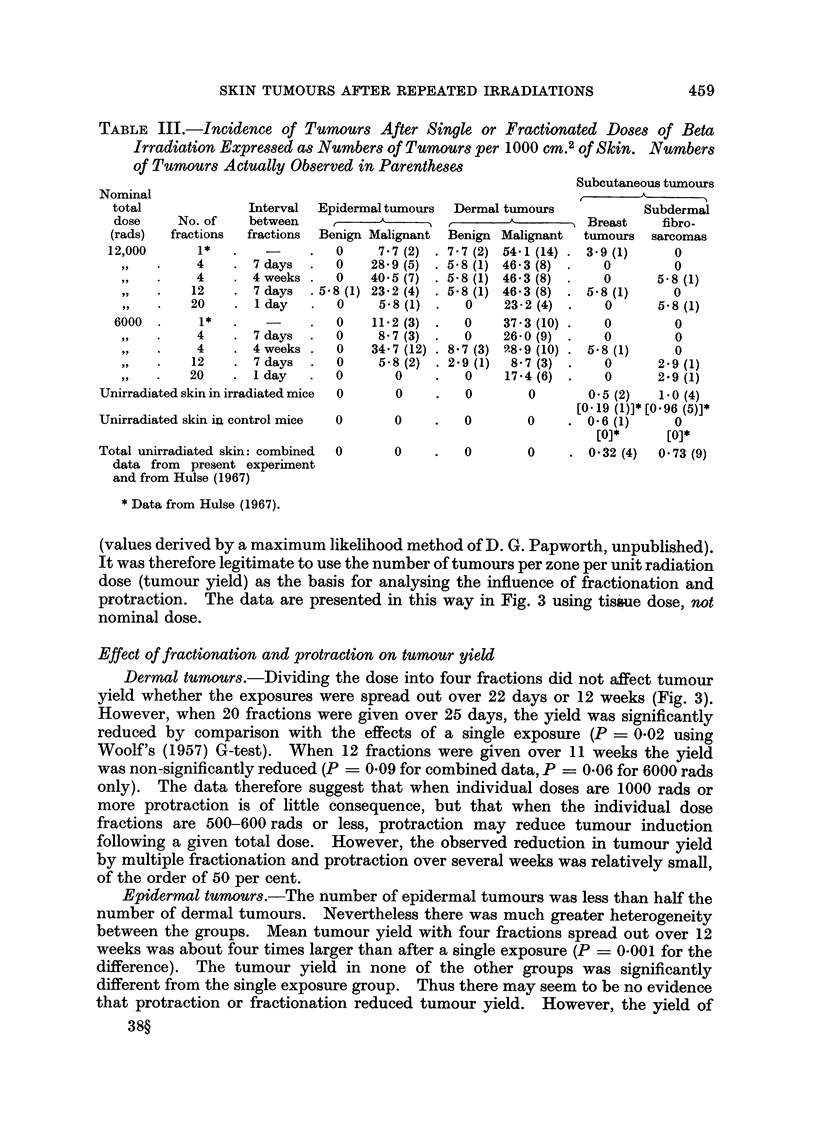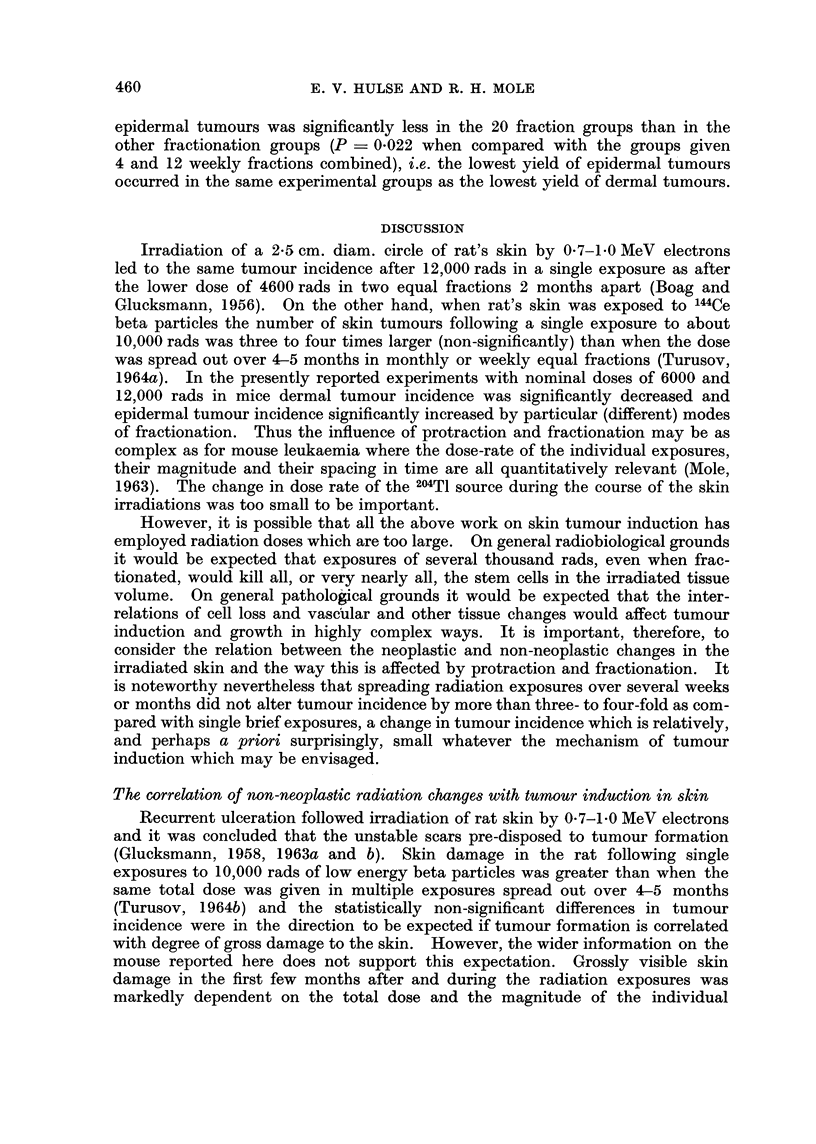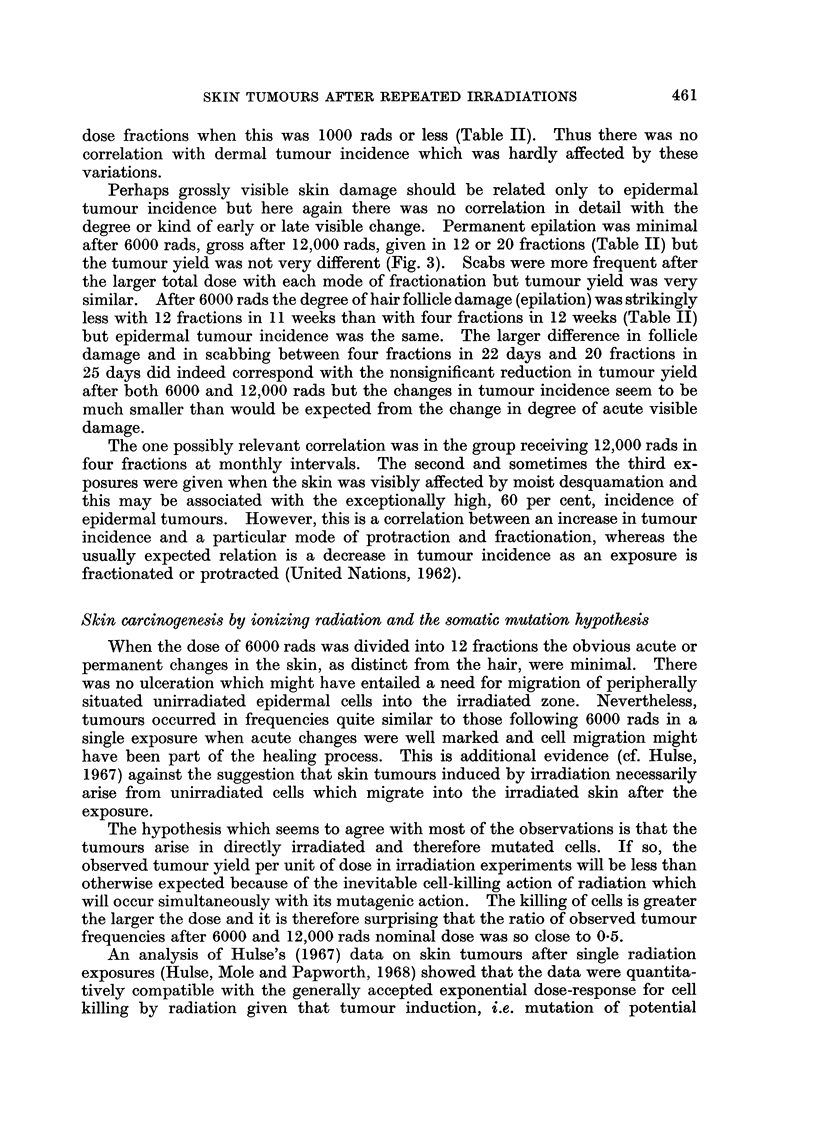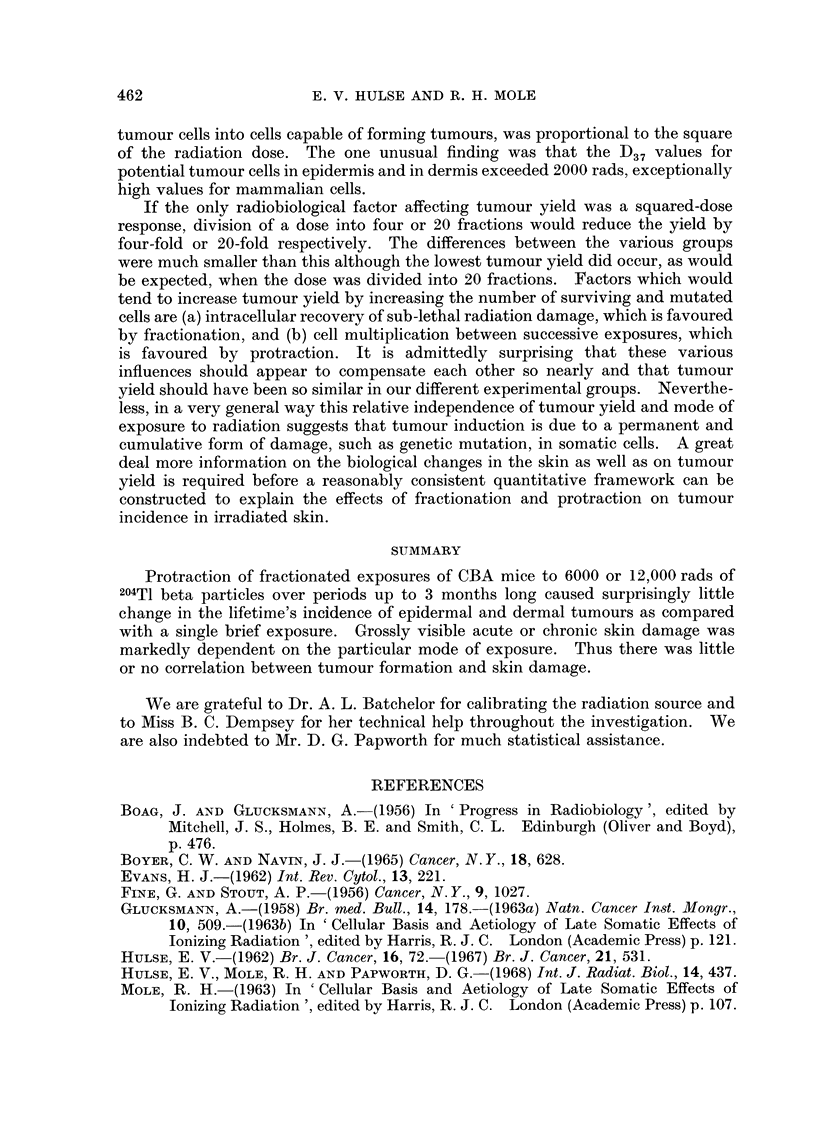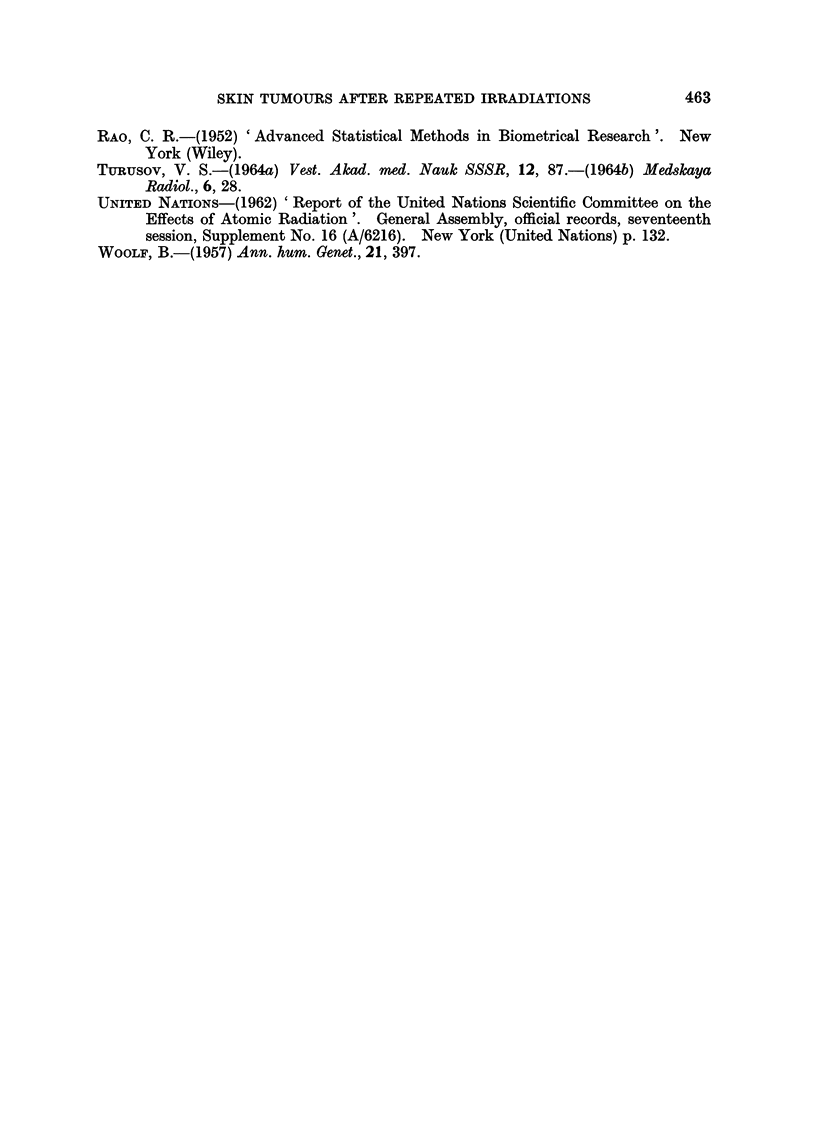# Skin tumour incidence in CBA mice given fractionated exposures to low energy beta particles.

**DOI:** 10.1038/bjc.1969.58

**Published:** 1969-06

**Authors:** E. V. Hulse, R. H. Mole


### SKIN TUMOUR INCIDENCE IN CBA MICE GIVEN FRACTIONATED EXPOSURES TO LOW ENERGY BETA PARTICLES

E. V. HULSE AND R. H. MOLE

*From the Medical Research Council, Radiobiological Research Unit, Harwell, Didcot, Berkshire*

Received for publication January 1, 1969

THE results of a number of workers show a considerable quantitative consistency in the frequency of tumours per unit of radiation dose following exposure of the skin to large doses of ionizing radiation (Hulse, 1962). When low as well as high doses of $^{204}$Tl beta particles were used, the dose-response suggested that tumour induction increased according to the square of the dose (Hulse, 1967; Hulse, Mole and Papworth, 1968). Since the dose-response for the yield of chromosome aberrations following low LET irradiation is curvilinear, usually with a marked squared-dose component (Evans, 1962), one possible implication of the skin tumour data is that tumour induction depends on chromosome breakage and re-arrangement. If so, protraction of the radiation exposure over a period of time should reduce tumour frequency in the same way as it reduces the frequency of chromosome aberrations (Evans, 1962). In fact there have been few experimental investigations of the way protraction or fractionation of radiation exposure modifies tumour yield in spite of the obvious practical as well as academic interest of such an enquiry.

This paper gives the results of irradiating the skin with four different schedules of exposure: four equal doses at weekly intervals, four equal doses at monthly intervals, 12 equal doses at weekly intervals, 20 equal doses 5 days weekly for 4 weeks. The results can be compared with those following a single brief exposure to the same total dose in mice of the same strain irradiated with the same $^{204}$Tl source (Hulse, 1967).

### METHODS

### *Animals*

Two hundred and forty female CBA/H mice were irradiated when 3 months old and 60 unirradiated mice were kept as controls. The different doses and radiation schedules were randomly allocated to litters rather than single mice because the irradiations were to be repeated. The mice of one litter were kept together from birth till death unless a tumour developed, in which case the affected mouse was housed separately. Abnormalities of the skin seen during the period of the irradiations were noted and a routine record of the condition of the skin was made at 6 weeks after the last irradiation and at death. Mice were allowed to die naturally unless they were moribund or the size of tumour or its degree of ulceration made it necessary to kill the animal.

### *Irradiations*

Exactly the same procedures were followed as before (Hulse, 1967). The zone of skin irradiated by beta particles corresponded in shape and size to the $^{204}$Tl source, *i.e.* an open ended cylinder 1·1 cm. in length and 8·6 cm.$^2$ in area. The source was always positioned over the middle of the torso and the same zone was irradiated on each occasion. When exposures were repeated the position of the mouse in relation to the source was intended to be the same but naturally was not in fact always identical. The radiation dose was measured at the inner surface of the celluloid tube which held each mouse in position during the irradiation. As $^{204}$Tl beta particles have a relatively low maximum energy of 0·765 MeV the germinative layer of the epidermis received 69–72 per cent and the dermis 40–70 per cent of this nominal dose (Hulse, 1967). Thus 12,000 rads nominal was taken to be equivalent to an epidermal dose of 8400 rads and a dermal dose of 6600 rads.

The total nominal dose to a zone was either 12,000 or 6000 rads. The number of fractions, the overall duration of exposure and the number of mice irradiated are given in Table I. The mice which received 20 equal doses were irradiated daily from Monday to Friday for 4 consecutive weeks. For the weekly exposures each litter was allocated to a particular day of the week and the radiation was given strictly at 7-day intervals. The monthly exposures were similarly arranged at 4-week intervals. All the exposures for one type of fractionation were completed before the next was started and were done in the following order: 20 daily exposures, 4 weekly exposures, 4 monthly exposures and 12 weekly exposures. During the 21 months over which these exposures were given the nominal dose rate decreased from 75 rads/min. to 57 rads/min. because of the natural decay of $^{204}$Tl. The nominal dose rate for the single exposures was 87–68 rads/min. (Hulse, 1967).

*Tumour incidence*

Tumour growth was very variable. The mice were examined regularly and a note was made when a definite tumour was first evident clinically. For the purposes of analysing age-specific tumour rates this time has been taken as the time of occurrence of a tumour.

The incidence of tumours in unirradiated skin was based on the 60 unirradiated mice and on the unirradiated skin of the irradiated mice. The combined control data from these mice and from others (Hulse, 1967) are given in Tables I and III. It was presumed, as previously, that the skin of the torso of an unirradiated mouse was equal in area to three irradiated zones and that the unirradiated skin of a mouse irradiated over one zone was equal in area to two zones.

### RESULTS

*Externally visible changes in the skin*

Non-neoplastic effects of the various exposure regimes are summarised in Table II. Permanent epilation of the whole irradiated zone, recorded at death, occurred in virtually all mice receiving a nominal dose of 12,000 rads. With 6000 rads, however, there were marked differences according to the exposure schedule. Complete epilation of the whole zone was reduced in frequency when the total dose was given in four equal fractions and did not occur at all when there were 12 or 20 fractions. Even patches of complete epilation were then uncommon. The number of fractions determined the degree of permanent change not the overall exposure time (Table II) and the determining factor was therefore

TABLE I.—*Number of Mice Exposed to Fractionated Doses of Either* 12,000 *rads or* 6000 *rads of Beta Particles, the Timing of the Exposures, the Life-span of Mice Without Skin Tumours and the Percentage of Mice Dying With Skin Tumours*

| No. of fractions | Interval between successive exposures | Overall exposure time, *i.e.* time interval between first and last exposures | No. of mice 12,000 rads | No. of mice 6000 rads | †Age at death of mice not having tumours of skin Mean ± S.E. (months) 12,000 rads | †Age at death 6000 rads | Mice with skin tumours per cent 12,000 rads | Mice with skin tumours per cent 6000 rads |
|---|---|---|---|---|---|---|---|---|
| 1* | — | — | 30 | 31 | 25 ± 2 | 26 ± 2 | 57 | 42 |
| 4 | 7 days | 21 days | 20 | 40 | 27 ± 3 | 24 ± 2 | 60 | 28 |
| 4 | 4 weeks | 12 weeks | 20 | 40 | 21 ± 3 | 24 ± 2 | 75 | 55 |
| 12 | 7 days | 11 weeks | 20 | 40 | 29 ± 2 | 28 ± 1 | 65 | 15 |
| 20 | 1 day (5 days weekly) | 25 days | 20 | 40 | 28 ± 3 | 29 ± 1 | 30 | 15 |
| Unirradiated | — | — | 60 | | 31 ± 1 | | 0 | |
| Mock irradiated* | — | — | 58 | | 29 ± 1 | | 0 | |

* From Hulse (1967).
† Time since start of irradiation plus 3 months.

the magnitude of the individual exposure which was smaller the larger the number of fractions into which the fixed total dose was divided.

Early changes in the skin were also modified by fractionation (Table II). Since they were recorded at a fixed time after the end of the period of radiation

TABLE II.—*Radiation Changes in the Skin* (*Frequency Per Cent*) *at* 6 *Weeks After Last Exposure and at Death*

| Number of fractions | Overall exposure time | Six weeks after last exposure: Scabs | 6000 rads Epilation: Complete | 6000 rads Epilation: Patchy | 12,000 rads† scabs | Epilation at death 6000 rads: Complete | Epilation at death 6000 rads: Patchy | Epilation at death 12,000 rads complete |
|---|---|---|---|---|---|---|---|---|
| 1 | 1 day | 29 | 100 | — | 80 | 100 | — | 100 |
| 4 | 22 days | 30 | 90 | 5 | 50 | 75 | 18 | 100 |
| 4 | 12 weeks | 0 | 25 | 70 | 30 | 90 | 8 | 90 |
| 12 | 11 weeks | 0 | 0 | 0 | 10 | 0 | 0 | 100 |
| 20 | 25 days | 0 | 0 | 8 | 15 | 0 | 10 | 95 |

† With one exception all mice showed complete epilation of irradiated zones.

exposure it is legitimate to compare only those fractionation regimes having the same overall exposure time. It is then clear that all the recorded changes (except epilation after 12,000 rads) were reduced in frequency by an increase in the number of fractions *i.e.* a reduction in the magnitude of an individual exposure. When a total dose of 12,000 rads was given in four fractions of 3000 rads 4 weeks apart moist desquamation was visible at the time the second and third exposures were being given (though not at the time of the fourth exposure). No moist desquamation was seen in the mice receiving the same total dose over the same overall time but in 12 weekly fractions each of 1000 rads.

No delayed ulceration of the skin occurred. After healing of any acute lesions the epilated skin remained unchanged except for some contraction in width of the irradiated zone (cf. Hulse, 1967). The only progressive change was depigmentation of the hair which was complete long before death in all irradiated zones, except in two mice receiving 6000 rads in 20 fractions where depigmentation was only partial even at 23–24 months after exposure.

*Causes of death other than skin tumours*

All mice were autopsied and dates of death noted. As in the experiments with single exposures (Hulse, 1967), causes of death other than skin tumours were very similar in irradiated and unirradiated mice. The mean age at death for each group of irradiated mice was slightly lower than that for the corresponding controls (Table I) and the difference was statistically significant in three instances in each of which there was a special reason. In the two groups receiving monthly exposures skin tumour incidence late in life was exceptionally high and, as few elderly mice did not have skin tumours, the mean age at death for mice dying from other causes was reduced. In the third instance, the group receiving 6000 rads in 4 weekly exposures, there were 4 mice dead from uncertain causes (? accidental) at 8–12 months of age: when these are excluded the mean age at death from other causes was $26 \pm 1$ months.

*Tumours*

The tumours were very similar to those seen after single exposures (Hulse, 1967). A total of 98 skin tumours were seen, all in irradiated skin. The dermal and epidermal tumours were not concentrated at the edges of the irradiated zones but, as after single exposures, appeared to originate within the irradiated areas.

*Epidermal tumours.*—Thirty-four were squamous cell carcinomas, one a highly keratinised sessile papilloma.

*Dermal tumours.*—All seven benign tumours were fibromas; 3 contained small amounts of bone. Fifty-four malignant tumours were fibrosarcomas, most well differentiated; 2 contained small amounts of bone. Occasionally, the histological picture suggested that the tumour had started as a fibroma and only later became malignant (as noted before Hulse, 1967). The two other malignant dermal tumours were unusual, a haemangioendothelioma, microscopically invasive, and an osteosarcoma with widespread metastases, a type of dermal tumour not seen before in our mice. It was impossible to tell whether this metastatic osteogenic tumour had started *de novo* as an osteosarcoma of extraskeletal soft tissue (Fine and Stout, 1956) or whether it arose as a malignant change in a patch of ossification in the irradiated dermis. Osteosarcoma of soft tissue can follow irradiation in man but is exceedingly rare (Boyer and Navin, 1965).

*Multiple skin tumours.*—In seven mice two anatomically separate tumours developed in one irradiated zone. Two mice had a fibroma and a fibrosarcoma, two a fibroma and a squamous cell carcinoma and two a fibrosarcoma and a squamous cell carcinoma. The mouse with the haemangioendothelioma also had a squamous cell carcinoma. In an eighth animal the superficial part of a neoplastic mass was a squamous cell carcinoma and the deeper part a fibrosarcoma. As in a similar example seen before (Hulse, 1967) there was no evidence of metaplasia from one type of tumour to the other.

Animals with two tumours were evenly distributed amongst the various groups in proportion to the total number of tumours and were not concentrated at any particular dose level or mode of exposure.

*Subdermal tumours.*—As previously (Hulse, 1967) a few subdermal tumours occurred, two breast tumours and four fibrosarcomas beneath irradiated skin and three breast tumours and four fibrosarcomas beneath unirradiated skin.

*The development of skin tumours with the lapse of time*

After single exposures 80 per cent of the mice carrying epidermal or dermal tumours died or were killed within 3 months of the record of first appearance. After fractionated exposures tumour growth seemed to be slower since only one-third of the mice with epidermal tumours and one-half of those with dermal tumours were dead within this period. Although the median time interval between appearance and death was less for dermal than for epidermal tumours, 3 months as against $4\frac{1}{2}$ months, a higher proportion of dermal tumours were very slow growing. The interval between appearance and death exceeded 7 months in over half the fibromas and in one eighth of the malignant dermal tumours but only in one single case of an epidermal tumour. No detected tumour ever regressed.

The two earliest tumours (one fibrosarcoma, one squamous carcinoma) were seen for the first time in the ninth month after the last of 4 monthly fractions of 3000 rads each. The first fibroma appeared 18 months after the last of 12 weekly exposures to the same total of 12,000 rads. Fig. 1 and 2 illustrate the age-specific rates of appearance of new dermal and epidermal tumours calculated for successive

FIG. 1.—Age specific incidence of dermal tumours after single or fractionated exposures to nominal doses of either 12,000 rads or 6000 rads of beta particles. The age-specific incidence is the ratio of number of tumours detected during a 6-month interval to number of mice alive at the beginning of the interval. The ratios were determined for successive non-overlapping 6-month intervals beginning at the time of the first radiation exposure and expressed as tumours per 1000 cm.$^2$ of irradiated skin. They are plotted at the middle of the 6-month period to which they refer.

——— Single dose

—●— 4 monthly fractions

—○— 4 weekly fractions

—▽— 12 weekly fractions

—▲— 20 daily fractions

FIG. 2.—Age-specific incidence of epidermal tumours after single or fractionated exposures to nominal doses of either 12,000 rads or 6000 rads of beta particles. Presentation as in Fig. 1.

——— Single dose

—●— 4 monthly fractions

—○— 4 weekly fractions

—▽— 12 weekly fractions

—▲— 20 daily fractions

6-month periods after the start of irradiation. There is no material difference when time is measured from the mid-point of the exposures. In general the peak tumour rate occurred in the second half of the second year or the first half of the third year after exposure whether the exposures were single or fractionated and whether tumours were dermal or epidermal. At later times the rates of appearance of new epidermal tumours decreased in all groups and of new dermal tumours decreased in six of the ten groups. It seems justifiable, taking into account the uniform survival time of mice without skin tumours, to take the total cumulative tumour incidence as a fair statistic for comparisons between groups. It is recognised that this may underestimate the real yield of dermal tumours after some modes of fractionation.

*Tumour Incidence*

The two earliest tumours appeared 12 months after the start of irradiation, *i.e.* at 15 months of age. Since mortality at that time was small, one to five in five of the eight groups given fractionated exposures, no correction for early mortality has been made and the reported incidences (Table I) are based on the original number of mice irradiated. The proportion of mice with one or more skin tumours ranged from 15 to 75 per cent. Since some mice carried more than one tumour, incidence is better expressed as numbers of tumour per mouse. As in previous reports of experiments in which only part of the skin was exposed (Hulse, 1962, 1967) incidence is given in numbers of tumours per 1000 cm.$^2$ of irradiated skin (Table III). Tumour yield is number of tumours per 1000 cm.$^2$ of skin per unit radiation dose (Fig. 3).

FIG. 3.—The yield of dermal and epidermal tumours after single or fractionated exposures to beta particles. The yield is given as tumours per mouse per 1000 rads tissue dose (see text). The standard errors were calculated assuming a Poisson distribution of tumours. The shaded columns represent data from mice given a total nominal air dose of 12,000 rads (average tissue doses: 8400 rads to the epidermis and 6600 rads to the dermis) and the unshaded columns those given a nominal 6000 rads (average tissue doses: 4200 rads to the epidermis and 3300 rads to the dermis). M means one exposure every 4 weeks, W every 7 days and D daily 5 days a week.

The overall incidence of subdermal tumours for 6000 and 12,000 rads combined was not significantly different from the control incidence. Benign dermal tumours were too few to allow meaningful comparisons between the various radiation regimes but their frequencies in all the irradiated groups combined, 3/80 after 12,000 rads and 4/160 after 6000 rads, were each significantly greater than in unirradiated skin ($P \leqslant 0{\cdot}0025$ by Rao's (1952) exact test). In all that follows benign and malignant skin tumours have been combined giving two main categories, epidermal and dermal, and the statistical tests are based on the assumption of a Poisson distribution of tumours among the irradiated zones. The ratio of epidermal to dermal tumours was 1 : 5 after single exposures, 1 : 2 after fractionated exposures (cf. Table III).

*Differences in tumour incidence according to dose*

The data in Table I suggest that tumour incidence was approximately half as great after 6000 rads as after 12,000 rads. Formal statistical tests using numbers of tumours per zone for the five sets of mice (four fractionated exposures, one single exposure) showed that the weighted ratio of incidences after 6000 and 12,000 rads was $0{\cdot}54 \pm 0{\cdot}17$ for epidermal and $0{\cdot}54 \pm 0{\cdot}12$ for dermal tumours

TABLE III.—*Incidence of Tumours After Single or Fractionated Doses of Beta Irradiation Expressed as Numbers of Tumours per* 1000 *cm.*$^2$ *of Skin. Numbers of Tumours Actually Observed in Parentheses*

| Nominal total dose (rads) | No. of fractions | Interval between fractions | Epidermal tumours | | Dermal tumours | | Subcutaneous tumours | |
|---|---|---|---|---|---|---|---|---|
| | | | Benign | Malignant | Benign | Malignant | Breast tumours | Subdermal fibro-sarcomas |
| 12,000 | 1* | — | 0 | 7·7 (2) | 7·7 (2) | 54·1 (14) | 3·9 (1) | 0 |
| ,, | 4 | 7 days | 0 | 28·9 (5) | 5·8 (1) | 46·3 (8) | 0 | 0 |
| ,, | 4 | 4 weeks | 0 | 40·5 (7) | 5·8 (1) | 46·3 (8) | 0 | 5·8 (1) |
| ,, | 12 | 7 days | 5·8 (1) | 23·2 (4) | 5·8 (1) | 46·3 (8) | 5·8 (1) | 0 |
| ,, | 20 | 1 day | 0 | 5·8 (1) | 0 | 23·2 (4) | 0 | 5·8 (1) |
| 6000 | 1* | — | 0 | 11·2 (3) | 0 | 37·3 (10) | 0 | 0 |
| ,, | 4 | 7 days | 0 | 8·7 (3) | 0 | 26·0 (9) | 0 | 0 |
| ,, | 4 | 4 weeks | 0 | 34·7 (12) | 8·7 (3) | 28·9 (10) | 5·8 (1) | 0 |
| ,, | 12 | 7 days | 0 | 5·8 (2) | 2·9 (1) | 8·7 (3) | 0 | 2·9 (1) |
| ,, | 20 | 1 day | 0 | 0 | 0 | 17·4 (6) | 0 | 2·9 (1) |
| Unirradiated skin in irradiated mice | | | 0 | 0 | 0 | 0 | 0·5 (2) [0·19 (1)]* | 1·0 (4) [0·96 (5)]* |
| Unirradiated skin in control mice | | | 0 | 0 | 0 | 0 | 0·6 (1) [0]* | 0 [0]* |
| Total unirradiated skin: combined data from present experiment and from Hulse (1967) | | | 0 | 0 | 0 | 0 | 0·32 (4) | 0·73 (9) |

* Data from Hulse (1967).

(values derived by a maximum likelihood method of D. G. Papworth, unpublished). It was therefore legitimate to use the number of tumours per zone per unit radiation dose (tumour yield) as the basis for analysing the influence of fractionation and protraction. The data are presented in this way in Fig. 3 using tissue dose, *not* nominal dose.

*Effect of fractionation and protraction on tumour yield*

*Dermal tumours.*—Dividing the dose into four fractions did not affect tumour yield whether the exposures were spread out over 22 days or 12 weeks (Fig. 3). However, when 20 fractions were given over 25 days, the yield was significantly reduced by comparison with the effects of a single exposure ($P = 0{\cdot}02$ using Woolf's (1957) G-test). When 12 fractions were given over 11 weeks the yield was non-significantly reduced ($P = 0{\cdot}09$ for combined data, $P = 0{\cdot}06$ for 6000 rads only). The data therefore suggest that when individual doses are 1000 rads or more protraction is of little consequence, but that when the individual dose fractions are 500–600 rads or less, protraction may reduce tumour induction following a given total dose. However, the observed reduction in tumour yield by multiple fractionation and protraction over several weeks was relatively small, of the order of 50 per cent.

*Epidermal tumours.*—The number of epidermal tumours was less than half the number of dermal tumours. Nevertheless there was much greater heterogeneity between the groups. Mean tumour yield with four fractions spread out over 12 weeks was about four times larger than after a single exposure ($P = 0{\cdot}001$ for the difference). The tumour yield in none of the other groups was significantly different from the single exposure group. Thus there may seem to be no evidence that protraction or fractionation reduced tumour yield. However, the yield of epidermal tumours was significantly less in the 20 fraction groups than in the other fractionation groups ($P = 0{\cdot}022$ when compared with the groups given 4 and 12 weekly fractions combined), *i.e.* the lowest yield of epidermal tumours occurred in the same experimental groups as the lowest yield of dermal tumours.

### DISCUSSION

Irradiation of a 2·5 cm. diam. circle of rat's skin by 0·7–1·0 MeV electrons led to the same tumour incidence after 12,000 rads in a single exposure as after the lower dose of 4600 rads in two equal fractions 2 months apart (Boag and Glucksmann, 1956). On the other hand, when rat's skin was exposed to $^{144}$Ce beta particles the number of skin tumours following a single exposure to about 10,000 rads was three to four times larger (non-significantly) than when the dose was spread out over 4–5 months in monthly or weekly equal fractions (Turusov, 1964*a*). In the presently reported experiments with nominal doses of 6000 and 12,000 rads in mice dermal tumour incidence was significantly decreased and epidermal tumour incidence significantly increased by particular (different) modes of fractionation. Thus the influence of protraction and fractionation may be as complex as for mouse leukaemia where the dose-rate of the individual exposures, their magnitude and their spacing in time are all quantitatively relevant (Mole, 1963). The change in dose rate of the $^{204}$Tl source during the course of the skin irradiations was too small to be important.

However, it is possible that all the above work on skin tumour induction has employed radiation doses which are too large. On general radiobiological grounds it would be expected that exposures of several thousand rads, even when fractionated, would kill all, or very nearly all, the stem cells in the irradiated tissue volume. On general pathological grounds it would be expected that the interrelations of cell loss and vascular and other tissue changes would affect tumour induction and growth in highly complex ways. It is important, therefore, to consider the relation between the neoplastic and non-neoplastic changes in the irradiated skin and the way this is affected by protraction and fractionation. It is noteworthy nevertheless that spreading radiation exposures over several weeks or months did not alter tumour incidence by more than three- to four-fold as compared with single brief exposures, a change in tumour incidence which is relatively, and perhaps *a priori* surprisingly, small whatever the mechanism of tumour induction which may be envisaged.

### *The correlation of non-neoplastic radiation changes with tumour induction in skin*

Recurrent ulceration followed irradiation of rat skin by 0·7–1·0 MeV electrons and it was concluded that the unstable scars pre-disposed to tumour formation (Glucksmann, 1958, 1963*a* and *b*). Skin damage in the rat following single exposures to 10,000 rads of low energy beta particles was greater than when the same total dose was given in multiple exposures spread out over 4–5 months (Turusov, 1964*b*) and the statistically non-significant differences in tumour incidence were in the direction to be expected if tumour formation is correlated with degree of gross damage to the skin. However, the wider information on the mouse reported here does not support this expectation. Grossly visible skin damage in the first few months after and during the radiation exposures was markedly dependent on the total dose and the magnitude of the individual dose fractions when this was 1000 rads or less (Table II). Thus there was no correlation with dermal tumour incidence which was hardly affected by these variations.

Perhaps grossly visible skin damage should be related only to epidermal tumour incidence but here again there was no correlation in detail with the degree or kind of early or late visible change. Permanent epilation was minimal after 6000 rads, gross after 12,000 rads, given in 12 or 20 fractions (Table II) but the tumour yield was not very different (Fig. 3). Scabs were more frequent after the larger total dose with each mode of fractionation but tumour yield was very similar. After 6000 rads the degree of hair follicle damage (epilation) was strikingly less with 12 fractions in 11 weeks than with four fractions in 12 weeks (Table II) but epidermal tumour incidence was the same. The larger difference in follicle damage and in scabbing between four fractions in 22 days and 20 fractions in 25 days did indeed correspond with the nonsignificant reduction in tumour yield after both 6000 and 12,000 rads but the changes in tumour incidence seem to be much smaller than would be expected from the change in degree of acute visible damage.

The one possibly relevant correlation was in the group receiving 12,000 rads in four fractions at monthly intervals. The second and sometimes the third exposures were given when the skin was visibly affected by moist desquamation and this may be associated with the exceptionally high, 60 per cent, incidence of epidermal tumours. However, this is a correlation between an increase in tumour incidence and a particular mode of protraction and fractionation, whereas the usually expected relation is a decrease in tumour incidence as an exposure is fractionated or protracted (United Nations, 1962).

*Skin carcinogenesis by ionizing radiation and the somatic mutation hypothesis*

When the dose of 6000 rads was divided into 12 fractions the obvious acute or permanent changes in the skin, as distinct from the hair, were minimal. There was no ulceration which might have entailed a need for migration of peripherally situated unirradiated epidermal cells into the irradiated zone. Nevertheless, tumours occurred in frequencies quite similar to those following 6000 rads in a single exposure when acute changes were well marked and cell migration might have been part of the healing process. This is additional evidence (cf. Hulse, 1967) against the suggestion that skin tumours induced by irradiation necessarily arise from unirradiated cells which migrate into the irradiated skin after the exposure.

The hypothesis which seems to agree with most of the observations is that the tumours arise in directly irradiated and therefore mutated cells. If so, the observed tumour yield per unit of dose in irradiation experiments will be less than otherwise expected because of the inevitable cell-killing action of radiation which will occur simultaneously with its mutagenic action. The killing of cells is greater the larger the dose and it is therefore surprising that the ratio of observed tumour frequencies after 6000 and 12,000 rads nominal dose was so close to 0·5.

An analysis of Hulse's (1967) data on skin tumours after single radiation exposures (Hulse, Mole and Papworth, 1968) showed that the data were quantitatively compatible with the generally accepted exponential dose-response for cell killing by radiation given that tumour induction, *i.e.* mutation of potential tumour cells into cells capable of forming tumours, was proportional to the square of the radiation dose. The one unusual finding was that the $D_{37}$ values for potential tumour cells in epidermis and in dermis exceeded 2000 rads, exceptionally high values for mammalian cells.

If the only radiobiological factor affecting tumour yield was a squared-dose response, division of a dose into four or 20 fractions would reduce the yield by four-fold or 20-fold respectively. The differences between the various groups were much smaller than this although the lowest tumour yield did occur, as would be expected, when the dose was divided into 20 fractions. Factors which would tend to increase tumour yield by increasing the number of surviving and mutated cells are (a) intracellular recovery of sub-lethal radiation damage, which is favoured by fractionation, and (b) cell multiplication between successive exposures, which is favoured by protraction. It is admittedly surprising that these various influences should appear to compensate each other so nearly and that tumour yield should have been so similar in our different experimental groups. Nevertheless, in a very general way this relative independence of tumour yield and mode of exposure to radiation suggests that tumour induction is due to a permanent and cumulative form of damage, such as genetic mutation, in somatic cells. A great deal more information on the biological changes in the skin as well as on tumour yield is required before a reasonably consistent quantitative framework can be constructed to explain the effects of fractionation and protraction on tumour incidence in irradiated skin.

### SUMMARY

Protraction of fractionated exposures of CBA mice to 6000 or 12,000 rads of $^{204}Tl$ beta particles over periods up to 3 months long caused surprisingly little change in the lifetime's incidence of epidermal and dermal tumours as compared with a single brief exposure. Grossly visible acute or chronic skin damage was markedly dependent on the particular mode of exposure. Thus there was little or no correlation between tumour formation and skin damage.

We are grateful to Dr. A. L. Batchelor for calibrating the radiation source and to Miss B. C. Dempsey for her technical help throughout the investigation. We are also indebted to Mr. D. G. Papworth for much statistical assistance.

### REFERENCES

Boag, J. and Glucksmann, A.—(1956) In ' Progress in Radiobiology ', edited by Mitchell, J. S., Holmes, B. E. and Smith, C. L. Edinburgh (Oliver and Boyd), p. 476.

Boyer, C. W. and Navin, J. J.—(1965) *Cancer, N.Y.*, **18**, 628.

Evans, H. J.—(1962) *Int. Rev. Cytol.*, **13**, 221.

Fine, G. and Stout, A. P.—(1956) *Cancer, N.Y.*, **9**, 1027.

Glucksmann, A.—(1958) *Br. med. Bull.*, **14**, 178.—(1963*a*) *Natn. Cancer Inst. Mongr.*, **10**, 509.—(1963*b*) In ' Cellular Basis and Aetiology of Late Somatic Effects of Ionizing Radiation ', edited by Harris, R. J. C. London (Academic Press) p. 121.

Hulse, E. V.—(1962) *Br. J. Cancer*, **16**, 72.—(1967) *Br. J. Cancer*, **21**, 531.

Hulse, E. V., Mole, R. H. and Papworth, D. G.—(1968) *Int. J. Radiat. Biol.*, **14**, 437.

Mole, R. H.—(1963) In ' Cellular Basis and Aetiology of Late Somatic Effects of Ionizing Radiation ', edited by Harris, R. J. C. London (Academic Press) p. 107.

RAO, C. R.—(1952) 'Advanced Statistical Methods in Biometrical Research'. New York (Wiley).
TURUSOV, V. S.—(1964*a*) *Vest. Akad. med. Nauk SSSR*, **12**, 87.—(1964*b*) *Medskaya Radiol.*, **6**, 28.
UNITED NATIONS—(1962) 'Report of the United Nations Scientific Committee on the Effects of Atomic Radiation'. General Assembly, official records, seventeenth session, Supplement No. 16 (A/6216). New York (United Nations) p. 132.
WOOLF, B.—(1957) *Ann. hum. Genet.*, **21**, 397.